# Evaluation of Serum Prolactin Levels in Intellectually Disabled Patients Using Antipsychotic Medications

**DOI:** 10.5812/ijem.4366

**Published:** 2012-12-21

**Authors:** Tammy L Lambert, Kevin C Farmer, Nancy C Brahm

**Affiliations:** 1 Department of Clinical and Administrative Sciences, College of Pharmacy, University of Oklahoma, Oklahoma City, USA

**Keywords:** Prolactin, Intellectually Disabled, Antipsychotic Agents

## Abstract

**Background:**

Patients with intellectual disabilities may be treated with antipsychotic medications for a variety of diagnoses. Use of this category of medication can increase prolactin levels and place the patient at risk for sexual dysfunction and lower bone mineral density. The proposed mechanism of action is affinity for the dopamine receptor. Use of bromocriptine, a dopamine receptor antagonist, was proposed to attenuate hyperprolactinemia.

**Objectives:**

The objectives of this study were to (1) review serum prolactin (PRL) elevations associated with the use of antipsychotic (AP) medications in an intellectually disabled adult population and (2) determine if any association existed between the level of elevation and AP used.

**Patients and Methods:**

Medical records for adult patients at two Oklahoma facilities for the intellectually disabled were reviewed to evaluate prolactin levels for individuals prescribed antipsychotics. A linear regression model was used to evaluate the relationship between prolactin levels with intellectual disability level, bromocriptine use, demographics, and antipsychotic.

**Results:**

73 (n = 53 males, n = 20 females) patients met criteria. The average age was 41.2 years. Nearly 70% of the patients had severe to profound levels of disability. 77% were prescribed second generation antipsychotics; 19% received first generation agents. Two variables, gender and bromocriptine use, were found to be significant predictors of prolactin levels. Mean prolactin level for females was 44 ng/mL (normal range: 4-30 ng/mL, males = 4-23 ng/mL). Patients who did not receive bromocriptine had mean levels of 23 ng/mL. No significant difference in prolactin levels was found for type of AP.

**Conclusions:**

Mean prolactin levels for females were significantly higher than for males. Both sexes were found to have higher-than-normal levels. Use of bromocriptine was associated with higher prolactin levels. In this population of patients, the type of AP used had no significance on prolactin levels.

## 1. Background

Patients with developmental disabilities often present with additional diagnoses that necessitate the use of antipsychotic (AP) medications. These medications are often placed into two broad categories – first generation (FGA) and second generation (SGA) antipsychotics. The older FGA oral medications include chlorpromazine, fluphenazine, haloperidol and thioridazine. At the time of this study, SGA, also known as atypical antipsychotics, included aripiprazole, clozapine, olanzapine, paliperidone, quetiapine, risperidone, and ziprasidone. The mechanism of action is similar for both categories. Both FGA and SGA agents work by antagonizing dopamine (DA) receptors in the brain (1, 2). The older medications have a greater affinity for all DA receptors and bind more tightly (3). Efficacy of the older medications has been associated with increased ability to elevate prolactin (PRL) levels(3, 4). Originally the propensity of SGAs to elevate PRL levels was considered low, but this was found to vary by agent(4). An open-label single center trial that included use of risperidone, olanzapine, and quetiapine found that risperidone significantly increased PRL levels in all patients as early as one week following initiation (5). This supported previously reported work that compared monotherapy with clozapine, olanzapine, quetiapine, and risperidone with the addition of a FGA. In each case, the addition of a FGA significantly elevated the PRL level (4). A positive correlation between the AP dose and the PRL elevation was found (6).

The role of PRL in both men and women is diverse. Elevated levels may be the result of physical exertion, medication use, pituitary adenomas, and/or renal failure (7). In women it is the hormone responsible for preparing the breast tissue for lactation after pregnancy (8). It can be detected in males at much lower levels than those seen in females. A normal PRL level for non-pregnant females is 2 – 29 ng/mL and for pregnant females is 10 – 209 ng/mL. For males a normal level is 2 – 18 ng/mL (9). Adverse effects of elevated PRL levels include amenorrhea, galactorrhea, decreased libido, and erectile dysfunction (2). A suspected relationship seen with hyperprolactinemia secondary to AP use is osteoporosis leading to fractures (10). This relationship is supported by a recent review of studies (11). A positive correlation between elevated PRL levels and decreased bone mineral density has been found for both men and women (12, 13).

Bromocriptine mesylate is a DA receptor agonist that inhibits pituitary secretion of PRL without effecting other hormones (14). It has approved indications of acromegaly, hyperprolactinemia, Parkinson’s disease and Type 2 diabetes mellitus. Use in psychiatry has been limited due to concerns that bromocriptine use for antipsychotic-induced hyperprolactinemia has the potential to exacerbate psychotic symptoms or extrapyramidal symptoms (EPS) but this was not found in a recent study (15).

Dopamine receptors in the hypothalamus are a critical component of the negative feedback system for hormones such as PRL which is released by the pituitary gland (8). When levels rise above what is necessary for normal physiological functioning, DA receptors in the hypothalamus are stimulated triggering the pituitary gland to decrease secretion of PRL. If DA receptors are blocked by APs, the hypothalamus continues to stimulate the pituitary gland to produce and secrete PRL. This continued secretion leads to hyperprolactinemia which results in decreased secretion of gonadotropin-releasing hormone from the hypothalamus. As a result, secretion of both luteinizing hormone and follicle-stimulating hormone are decreased. In response to this action, the pituitary gland decreases release of the sex hormones which are responsible for normal bone metabolism.

Intellectually disabled adults have limited verbal communication abilities (16) which results in their inability to express a subjective experience of adverse drug reactions and side effects of medications. Prolonged PRL elevation has the potential to result in osteoporosis leading to bone fractures (17). Pain from a fracture may manifest itself in behaviors that may be interpreted as increases in aggression or elevation of psychotic behavior. This interpretation of a patient’s behavior could lead to increasing AP medication dosing while ignoring the actual physical problem of the fractured bone and pain.

## 2. Objectives

The objectives of this study were to (1) review serum PRL elevations associated with the use of AP medications in an intellectually disabled adult population and (2) determine if any association existed between the level of elevation and AP used.

## 3. Patients and Methods

Institutional Review Board (IRB) approval was obtained from the University of Oklahoma Health Sciences Center IRB. Confidentiality was maintained for all subjects. Pharmacy students on experiential rotations examined the medical records of adult patients at two Oklahoma facilities for people with intellectual disabilities for the presence of routine PRL levels. In addition, faculty responsible for students reviewed the findings. This examination resulted in 287 observations of patients having at least one PRL level drawn. These observations represent 73 different patients since some patients had serial PRL levels drawn.

The data collected from the patient medical records included name, medical record number, facility location, intellectual disability level consistent with criteria from the Diagnostic and Statistical Manual of Mental Disorders, fourth edition, text revision (DSM-IV-TR),(18) date of birth, current age, weight, gender, name (s) of antipsychotic medication, PRL level (s) and the date (s) of the level (s). Some patients were prescribed bromocriptine 2.5 mg twice daily. The decision to prescribe bromocriptine was made by the clinician when PRL levels were excessive. While the use of this medication was noted during data collection for this observational study, no information was provided to indicate the criteria used for prescribing the medication.

Intellectual disability level was categorized on a 5 point scale. The levels starting at 0 were borderline, mild, moderate, severe, severe-profound and profound. If no level was indicated in the medical records the level was labeled “unspecified.”

The AP medications that were prescribed to the patients included risperidone, olanzapine, quetiapine, chlorpromazine, fluphenazine, haloperidol, ziprasidone, clozapine, and thioridazine. For patients with multiple PRL levels, the average of the levels was used. Since the levels were drawn over the course of several years, the age of the patient when the first lab was drawn was used for analysis. This age was identified as baseline age. One laboratory was contracted to perform PRL levels during the study period. All PRL levels in this study were measured using the Prolactin Enzyme Immunoassay produced by Linear Chemicals, S.L (19). This assay employs the ELISA test. The minimal concentration of PRL that can be detected is approximately 1.0 ng/Ml (19). Using a linear regression model, the relationship between PRL levels and 5 variables was examined. The 5 variables were intellectual disability level, whether the patient received bromocriptine mesylate, baseline age, gender and AP medication(s). Data were analyzed using SPSS version 17. (SPSS, version 17. SPSS Inc., an IBM Company Headquarters, 233 S. Wacker Drive, 11th floor Chicago, Illinois 60606).

## 4. Results

Most patients (72.6%) were males with a mean baseline age of 41.2 years with a profound disability level (46.6%). Risperidone was the most commonly prescribed AP and utilized by 60.3% of the patients. A list of the AP medications used is contained in Table 1.

**Table 1:** Patient Data

	All Subjects	Males, No.(%)	Females, No.(%)
**Observations**	73	53 (72.6)	20 (27.4)
**Antipsychotic Medication**			
Risperidone	44 (60.3%)	31 (58.5)	13 (65)
Haloperidol	9	6 (11.3)	3 (15)
Olanzapine	7	5 (9.4)	2 (10)
Chlorpromazine	3	3 (5.7)	0
Quetiapine	3	3 (5.7)	0
No Drug	3	2 (3.77)	1 (5)
Fluphenazine	2	2 (3.8)	0
Ziprasidone	2	1 (1.9)	1 (5)
**Intellectual Disability Level **(18)^a^			
Borderline	1	1 (1.9)	0
Mild	13	9 (17)	4 (20)
Moderate	6	6 (11.3)	0
Severe	17	13 (24.5)	4 (20)
Profound	34 (46.6%)	23 (43.4)	11 (55)
Unspecified	2	1 (1.9)	1 (5)
**Mean Baseline Age**	41.2 years	41.2 years	41.1 years

^a^ Reference No. 18

Individuals prescribed risperidone were found to have the highest mean PRL levels compared to other SGAs and FGAs ( Table 2 ). Much higher mean PRL levels were also found in patients prescribed adjunctive bromocriptine treatment in addition to their antipsychotic medication (45.8ng/mL vs. 23 ng/mL).

**Table 2:** Mean Prolactin Levels

	Observations	Mean Prolactin Level, ng/mL (SD) a
**All Subjects**	73	33.1 (21.1)
**Males**	53	29 (16.1)
**Females**	20	44 (28.4)
**Intervention**		
**Yes**	26	45.8 (20.6)
**No**	22	23 (19.3)
**Unknown**	25	28.9 (16.9)
**Medication**		
**Risperidone**	44	35.9 (20.7)
**First Generation Antipsychotics**	14	33.2 (23.3)
**Other Second Generation Antipsychotics**	12	27 (20.9)
**No Drug**	3	14.9 (2.0)

^a^SD, Standard deviation

Linear regression analysis found only two statistically significant predictors of PRL level: gender, and use of bromocriptine were. The use of a particular AP was not a significant predictor of PRL level.

The relationship of PRL level with specific AP use was also examined. While the mean PRL level for patients taking risperidone (35.9 ng/mL) was higher than the levels of patients on other medications, this difference was not statistically significant. No difference was found when the medications were compared individually or when risperidone was compared to other SGAs or to FGAs. Patients receiving a SGA other than risperidone were grouped into a single group for two reasons. First, only three different SGAs were prescribed (olanzapine, quetiapine and ziprasidone) to 12 patients. Second, previous research has shown that risperidone acts on PRL elevation in a manner similar to FGAs (3, 10, 20, 21).

## 5. Discussion

The PRL levels associated with individuals on APs for both males and females were much higher than normal expected levels. Since bromocriptine was given to lower excessive PRL levels, it was expected that patients who had received bromocriptine would have lower PRL levels than those patients who had not received bromocriptine. However, patients receiving bromocriptine actually had higher PRL levels than those who did not. There are several possible explanations for this finding. First, only patients who were considered to have extremely high PRL levels may have been given bromocriptine. Bromocriptine may have lowered the initial PRL level but did not bring the level down to the normal range. Some researchers have speculated that extreme elevations of PRL may require higher doses of bromocriptine than were used in this study (2.5 mg twice daily)(15).

The second consideration addresses the potential physiological effects of concurrent administration of bromocriptine and an AP. Dopamine possesses a number of physiological effects. Receptor affinity and an appropriate level of DA receptor blockade are important requirements for AP action. The mechanism of action for AP agents, particularly those considered SGA, includes D₁ and D₂ receptor blockade. In the hypothalamo-hypophysial axis DA has been found to be the primary physiological inhibitor of PRL secretion. Since bromocriptine, a dopaminergic agonist, suppresses PRL, it has been proposed that concurrent use has the potential to blunt the benefits of AP use. One of the theoretical concerns for clinicians is that since bromocriptine antagonizes the effects of the APs, patients who were prone to aggressive outbursts when under-medicated may not have been given bromocriptine regardless of their PRL level (15).

Lastly, we were unable to determine conclusively from the medical record (34% of the patients in the study) whether they had received bromocriptine or not. The mean PRL level for this group was significantly different from patients who had received bromocriptine but was not significantly different from the non-bromocriptine group.

## 6. Limitations

This study has several limitations. The data were retrospective and observational only. A chart review was performed to collect the data. The relationship between the time of the lab work for the PRL level and the dosing interval of the AP was not specified in the medical records. There were also several pieces of information that may have been valuable to the research but were unavailable for this study. Information related to the AP including dose, history of medication use, length of treatment and changes in dosing was not available. Finally, the criteria or standards that were used to determine if and when bromocriptine was prescribed also was not provided.

The limited communication abilities of the intellectually disabled population led to additional limitations. These patients are primarily non-verbal and staff must rely on their personal interpretation of the source of patient behavior changes. Additionally, there is the ethical concern of conducting research on this vulnerable population.

## 7. Conclusions

Significant difference in PRL levels were not found between the different APs. Additional research in a larger sample may provide additional information. Another area that deserves further study is the examination of the relationship between the dose of the AP and the resultant PRL level. Ultimately, research should investigate the long-term effects of elevated PRL levels which may include bone mineral density problems leading to fractures. The extensive use of APs in the intellectually disabled population in combination with high PRL levels may predispose this population to a higher risk of bone fractures. This is complicated with the difficulties of these individuals in communicating pain and discomfort symptoms to health care providers which may result in a lack of treatment or a misdiagnosis of the resultant behaviors exhibited by these individuals.

## References

[1] Molitch ME (2008). Drugs and prolactin.. Pituitary..

[2] Molitch ME (2005). Medication-induced hyperprolactinemia.. Mayo Clinic Proceedings..

[3] Maguire GA (2002). Prolactin elevation with antipsychotic medications: mechanisms of action and clinical consequences.. J Clin Psy..

[4] Montgomery J, Winterbottom E, Jessani M, Kohegyi E, Fulmer J, Seamonds B (2004). Prevalence of hyperprolactinemia in schizophrenia: association with typical and atypical antipsychotic treatment.. J Clin Psy..

[5] Svestka J, Synek O, Tomanova J, Rodakova I, Cejpkova A (2007). Differences in the effect of second-generation antipsychotics on prolactinaemia: six weeks open-label trial in female in-patients.. Neuro Endocrinol Lett..

[6] Smith S, Wheeler MJ, Murray R, O'Keane V (2002). The effects of antipsychotic-induced hyperprolactinaemia on the hypothalamic-pituitary-gonadal axis.. J Clin Psychopharmacol..

[7] Bolyakov A, Paduch DA (2011). Prolactin in men's health and disease.. Current opinion in urology..

[8] Haddad PM, Wieck A (2004). Antipsychotic-induced hyperprolactinaemia: mechanisms, clinical features and management.. Drugs..

[9] National Libraray of Medicine/National Institutes of Health: MedlinePlus.. http://www.nlm.nih.gov/medlineplus/ency/article/003718.htm.

[10] Kishimoto T, Watanabe K, Shimada N, Makita K, Yagi G, Kashima H (2008). Antipsychotic-induced hyperprolactinemia inhibits the hypothalamo-pituitary-gonadal axis and reduces bone mineral density in male patients with schizophrenia.. Journal of Clinical Psychiatry..

[11] Crews MPK, Howes OD (2012). Is antipsychotic treatment linked to low bone mineral density and osteoporosis? A review of the evidence and the clinical implications.. Human Psychopharm Clin Exper..

[12] Greenspan SL, Neer RM, Ridgway EC, Klibanski A (1986). Osteoporosis in men with hyperprolactinemic hypogonadism.. Annals of internal medicine..

[13] Klibanski A, Neer RM, Beitins IZ, Ridgway EC, Zervas NT, McArthur JW (1980). Decreased bone density in hyperprolactinemic women.. The New England journal of medicine..

[14] (2011). Bromocriptine mesylate [database on the Internet]. Facts & Comparison eAnswers [online]..

[15] Lee MS, Song HC, An H, Yang J, Ko YH, Jung IK (2010). Effect of bromocriptine on antipsychotic drug-induced hyperprolactinemia: eight-week randomized, single-blind, placebo-controlled, multicenter study.. Psy Clin Neur..

[16] Rush KS, Bowman LG, Eidman SL, Toole LM, Mortenson BP (2004). Assessing psychopathology in individuals with developmental disabilities.. Behav Modif..

[17] Stubbs B (2009). Antipsychotic-induced hyperprolactinaemia in patients with schizophrenia: considerations in relation to bone mineral density.. J Psychiatr Ment Health Nurs..

[18] Kassirer JP (1995). The next transformation in the delivery of health care.. New England Journal of Medicine..

[19] Prolacting Enzyme Immunoassay Test Kit.

[20] Becker D, Liver O, Mester R, Rapoport M, Weizman A, Weiss M (2003). Risperidone, but not olanzapine, decreases bone mineral density in female premenopausal schizophrenia patients.. J Clin Psy..

[21] David SR, Taylor CC, Kinon BJ, Breier A (2000). The effects of olanzapine, risperidone, and haloperidol on plasma prolactin levels in patients with schizophrenia.. Clin Ther..

